# Trends and implications of 24/7 interventional radiology in a newly opened acute hospital

**DOI:** 10.1186/s42155-018-0033-1

**Published:** 2018-10-29

**Authors:** Raymond Chung, Ashish Chawla, Sumer Shikhare, Suresh Babu

**Affiliations:** Department of Diagnostic Radiology at Khoo Teck Puat Hospital, 90 Yishun Central, Singapore, 768828 Singapore

**Keywords:** Interventional radiology, Out of hours, On call

## Abstract

**Introduction:**

We aim to evaluate the frequency of use and changing practices for all out-of-hours interventional radiology (IR) procedures performed in a new hospital.

**Methods:**

This is a 5 year retrospective review of all out-of-hours procedures performed by the Interventional Radiology team from July 2010 to June 2015. Number and category of procedures performed were identified from the RIS database.

**Results:**

Of the 7140 procedures performed by IR over the 5 years, 764 were out-of-hours. The total number of out-of-hours cases performed annually by IR has increased by 240% from year 1 to year 5. The variety and distribution of out-of-hours work has shown a characteristic trend with rising requests for advanced procedures such as active haemorrhage control.

**Conclusion:**

The rising number and complexity of cases for on-call IR further supports the need for a formal on-call rota, ideally 1:6, to provide a sustainable 24/7 service and optimize patient outcome in an acute hospital.

## Introduction

Interventional radiology (IR) is widely recognized as a pivotal clinical service in the management of emergency patients (Lerner et al., 2014) with a large evidence base for IR services’ contribution to improved patient outcomes and safety. The acute pathologies suitable for intervention are broad, from basic procedures such as drainage to time critical haemorrhage control. CIRSE guidelines have also strongly stipulated the need for 24-h IR availability in the endovascular treatment of traumatic haemorrhage (Chakraverty et al., 2012).

Royal College of Radiology (RCR) guidelines and the Cardiovascular and Interventional Radiological Society of Europe (CIRSE) recommendations are that out-of-hours (OOH) IR should be subject to a formal rota, to ensure safe and reliable service provision (The Royal College of Radiologists, 2017; Tsetis et al., 2016; The Royal College of Radiologists, 2014). In reality, implementation is difficult both in availability of manpower and resources, with a national survey of interventional radiology provision across England in 2014 reporting a formal OOH provision of 68%: specifically, OOH provision for nephrostomy of 65.6%, endovascular intervention of 77.8%, embolization for post partum haemorrhage of 59.8% and general embolization services of 67.4% (NHS Improving Quality, 2014). Many authors cite the need for strategic planning of 24/7 IR services (NHS Improving Quality, 2014; Zealley et al., 2012).

Singapore’s total population has been and continues to grow, totalling 5.47 million in June 2014. Demand in healthcare is projected to rise alongside its enlarging and increasing ageing population. Since 2011, an extra 1700 acute beds and 1200 community hospital beds have been added; with approximately 590 of those being from our current institute serving more than 700,000 people living in the northern sector of Singapore. To the authors knowledge, there is a lack of data regarding the use of interventional radiology out of hours service in a completely new public hospital serving a large community providing first world medical care. Therefore, this study reports the frequency of use and types of procedures performed annually by our unit over the last 5 years. The implications for a well supported IR team to provide such a service is also discussed.

## Methods

This is a retrospective, 5-year, single-centre study performed at an acute hospital with trauma response facilities serving a neighbouring population of over 700,000. The hospital has been opened for just over 8 years, since July 2010. Since inception, there has been a separate IR on-call rota, starting with a single permanent interventional radiologist providing the dominant 24-h cover. A smaller percentage of the on-call cover was provided by “Visiting Consultants” who held dual positions in the private and public sector. From November 2014 to the end of the study, there were 4 full-time interventional radiologists supporting a two-tier on call system. Three provided a first on-call position, therefore working a 1 in 3 rota. Senior interventional radiology opinion was sought if required from the 4th IR Consultant. The remaining IR team members, consisting of 2 IR nurses and a dedicated IR radiographer, also follow a formal on-call rota.

All out-of-hours cases were identified via the hospital RIS database. Data was collated according to a full calendar year from 1st July to the 30th June of the next year. Inclusion criteria was purely time based, incorporating all procedures started outside of the normal working hours of 0800–1730 on a weekday, and all cases performed on the weekend or public holidays. Microsoft Excel® was used for data management and analysis. Procedures were classified as per Table 1.

**Table 1:** Classification of procedures, numbers of out-of hours performed annually and in total over 5 years

YEAR	Drainage	Vascular access	Thoracic angiography / embolization	Abdominopelvic angiography / embolization	Nephrostomy	Diagnostic neuroangiography	Peripheral vascular intervention	IVC filter	PTC	Visceral arterial stenting	Ureteric stent	TIPSS / BRTO	EVAR	Pulmonary thrombectomy
2010–2011	33	15	1	7	22	1	6	0	0	0	0	0	0	0
2011–2012	52	22	2	8	13	4	3	3	0	0	0	0	1	0
2012–2013	83	33	1	19	16	6	2	3	4	1	2	0	0	0
2013–2014	85	28	7	27	23	8	7	6	5	0	0	1	0	0
2014–2015	84	27	5	35	27	5	9	7	1	0	1	1	1	1
TOTAL	337	125	16	96	101	24	27	19	10	1	3	2	2	1

## Results

**Fig. 1:**
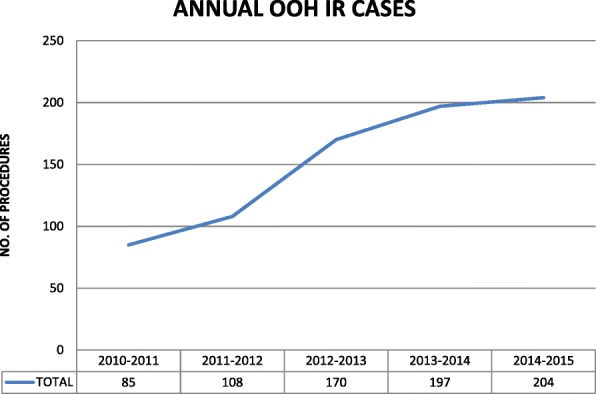
Total annual number of out-of-hours IR cases

Total annual number of out-of-hours cases has increased progressively throughout the 5 years from 85 to 204 (Fig. 1); as has the total number of IR procedures performed within the department (Table 2). The out-of-hours work as a percentage of all procedures performed has increased from 8% to 11–12%.

**Table 2:** Number of out-of-hour compared to total number of IR procedures performed in the unit

YEAR	No. IR OOH procedures	Total no. IR procedures	% OOH
2010–2011	85	995	8.5
2011–2012	108	1178	9.1
2012–2013	170	1504	11.3
2013–2014	197	1639	12.0
2014–2015	204	1824	11.2
TOTAL	764	7140	

The distribution of work throughout the week has also changed over the last 5 years (Table 3). The percentage of work being performed on the weekends has increased from 27.1% to 40.2%. Conversely, the percentage of cases performed out-of-hours on the weekdays has reduced from 65.9% to 56.9%.

**Table 3:** Distribution of out-of-hours cases performed through the week

	2010–2011	2011–2012	2012–2013	2013–2014	2014–2015
Weekday	65.8%	58.3%	68.2%	59.4%	56.9%
Weekend	27.1%	28.7%	27.6%	36.5%	40.2%
Public Holiday	7.1%	13.0%	4.2%	4.1%	2.9%

**Fig. 2:**
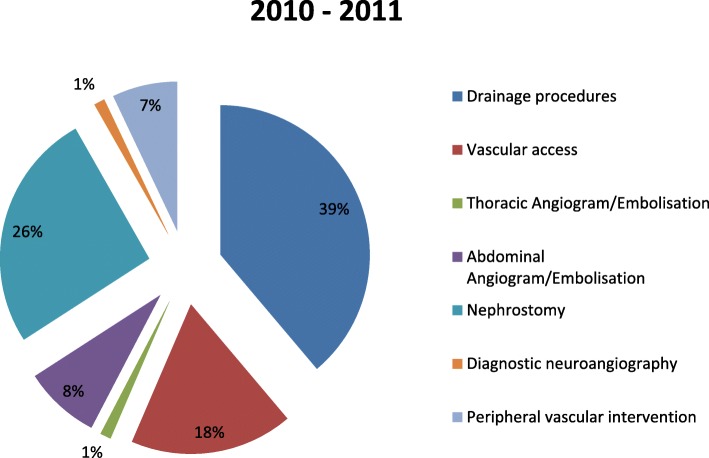
Procedures performed as a percentage in year cycle 2010–2011

**Fig. 3:**
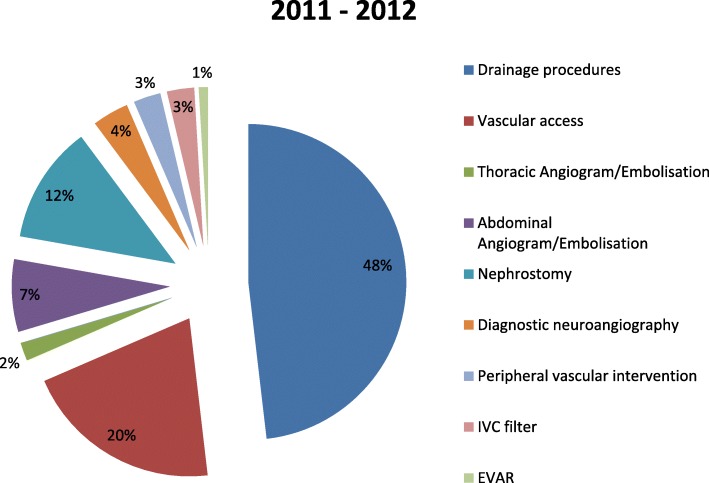
Procedures performed as a percentage in year cycle 2011–2012

**Fig. 4:**
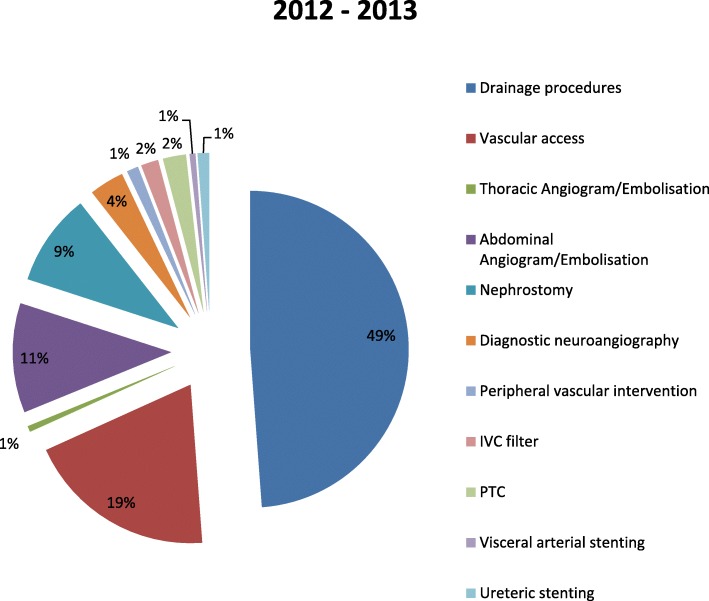
Procedures performed as a percentage in year cycle 2012–2013

**Fig. 5:**
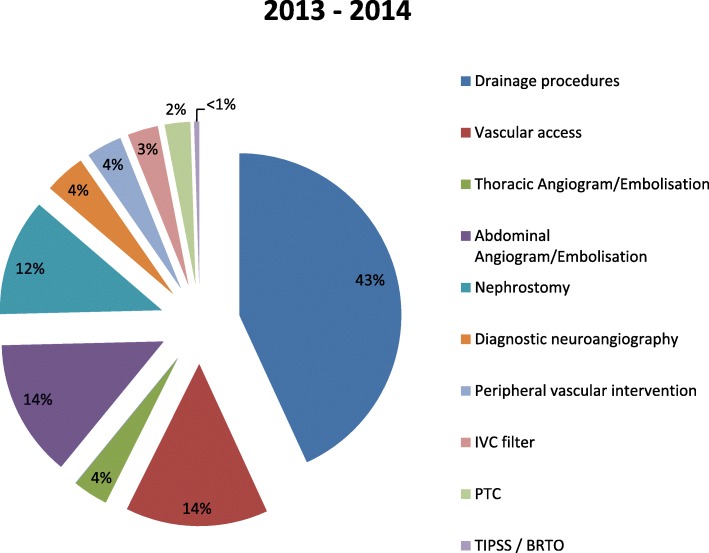
Procedures performed as a percentage in year cycle 2013–2014

**Fig. 6:**
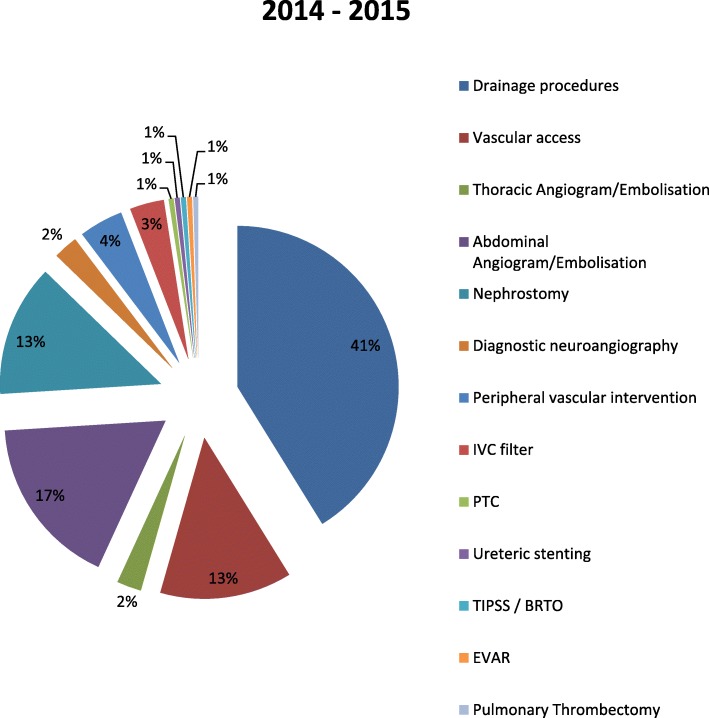
Procedures performed as a percentage in year cycle 2014–2015

The number of the procedures performed is shown in Table 1; and percentage contribution of each procedural subcategory in the respective year is illustrated in Figs. 2, 3, 4, 5, 6. Drain insertions have formed the majority of the workload throughout the years, at 41% in the past year. The 2nd and 3rd most common procedures have however demonstrated a change; with earlier years these were predominantly for either nephrostomy insertions or vascular access related procedures. The last 3 years have shown a steady increase of abdominopelvic angiogram and embolization procedures, which is now the 2nd most commonly performed out-of-hours procedure. Nephrostomy insertions and vascular access procedures are approximately joint 3rd in frequency performed.

The variety and complexity of procedures have also increased throughout the years, from 7 to 13 sub-groups; now including advanced techniques of TIPSS/BRTO, EVAR and pulmonary thrombectomy.

## Discussion

IR is playing an ever increasing role in the acute management of pathologies presenting across the clinical specialties both as the primary and secondary line of treatment (Ierardi et al., 2015). Both recent guidelines and standards of practice highlight the importance of having an interventional radiology team who are able to respond to a wide variety of clinical scenarios (Chakraverty et al., 2012; The Royal College of Radiologists, 2017).

With the rapidly expanding healthcare provision across Singapore, including both recent and future planned acute medical hospital openings, a well-trained, dedicated and certified IR team is a necessity for the delivery of first world medical care. Our data, as would be expected for a new hospital has shown a progressive increase in volume of work. In part, however, we suspect the availability of our team has altered the referral pathway and therapeutic regimes of emergency patients, and thus contributed to the increased workload. This is contrary to a recent study spanning 3.5 years across 11 hospitals offering a formal out-of-hours IR service covering a population of 1.2 million that concluded there was no evidence of expansion of demand despite availability (Christie et al., 2013). The increased volume of out-of-hours work, 240% from the 1st compared to the 5th year, arguably impacts the workflow pattern of the on-call interventionalists.

Recommendations are for a minimum of 6 radiologists to provide a comprehensive 24/7 IR service (The Royal College of Radiologists, 2017; Tsetis et al., 2016; The Royal College of Radiologists, 2014), with appropriate provisions for next-day radiology cover in the event of overnight cases, to allow sufficient rest and avoid compromise to patient safety. Our team has grown from a single interventionalist to 4, with a dedicated trained specialist IR nursing and radiography team. As demonstrated by Goltz et al. (Goltz et al., 2014) and in our study, we have managed to provide a 24/7 IR service with 3 first on-call radiologists. This partly reflects the geography of the city state, in which travel to the hospital is manageable within 45 min from most parts of the island, thus allowing an off-site on-call system. However, long-term sustainability based on 3 interventionalists is difficult and networking solutions with nearby units would allow a more healthy rota of 1:6 for a population of < 1 million (Ierardi et al., 2015; Lerner et al., 2014). In addition, a cascade system of workflow led by our specialist trained nurses allows a rapid and smooth patient pathway from point of acceptance of referral by the Interventional Radiologist on-call, to the IR procedure and subsequent discharge back to the clinical team’s care. This may serve as a benchmark for newer units with potentially larger capacity for their expected workload projections and implications of this for their IR teams.

In tandem with the rising volume of cases, we have experienced increasing complexity of interventional procedures requested and performed. The increase in abdominopelvic angiography and embolization procedures reflects our institute’s development of an acute trauma unit and IR’s subsequent role in their management. A fully supportive IR team is critical to avoid the reported differing standards of care and outcome experienced by a patient dependent upon time of presentation to hospital (Schwartz et al., 2014). Despite the relatively few number of interventionalists (4 interventional radiologists vs 23 diagnostic radiologists), it should not impede initiation or continuation of a 24/7 service. In our unit, since the introduction of a two-tier on-call system, > 95% of the cases can be handled by the first on-call interventional radiologist. Only a much smaller percentage of cases require the further expertise of our more seasoned interventionalist.

Our unit has also experienced a greater proportion of the work being performed during the weekends; 40.2% in 2015 compared to 27.1% in 2010. This probably reflects an increasing preferential utilization of IR services during the weekend and, our flexible and responsive action in-hours.

In this study, we included all cases that were started out-of-hours. This therefore did not include any urgent emergency cases performed within regular working hours or those cases extending beyond the regular working day. As we were proposing to study the utility of out-of-hours IR work, cases that may be deemed neither life nor limb threatening were also included such as those that were delayed due to accommodation of emergency cases. Nevertheless, a responsive IR team can undoubtedly benefit patient’s care and overall satisfaction, preventing prolonged in-hospital stays.

## Conclusion

Our single-centre data incorporating all out-of-hours cases performed over the last 5 years since inception has demonstrated a progressive increase in volume and complexity of interventional procedures and, provides a benchmark for newly opened and planned hospitals. Drainage procedures have formed the majority of the work, although abdominopelvic angiography and embolization procedures have shown a steady increase. A sufficient number of trained interventionalists, ideally a 1:6 rota, with sufficient free time compensation is recommended to support a sustainable 24/7 IR clinical service in an acute hospital.

## Abbreviations

BRTO: Balloon-occluded retrograde tranvenous obliteration
CIRSE: Cardiovascular and Interventional Radiological Society of Europe
EVAR: Endovascular aneurysm repair
IR: Interventional radiology
OOH: Out-of-hours
RCR: Royal College of Radiology
TIPSS: Transjugular intrahepatic portosystemic shunt
